# Crystal structure of {bis­[2-(3,5-di­methyl­pyrazol-1-yl-κ*N* ^2^)eth­yl]amine-κ*N*}chlorido­platinum(II) chloride dihydrate^1^

**DOI:** 10.1107/S2056989015005307

**Published:** 2015-03-21

**Authors:** María de los Angeles Mendoza, Sylvain Bernès, Guillermo Mendoza-Díaz

**Affiliations:** a Departamento de Ingenierías Química Electrónica y Biomédica, División de Ciencias e Ingenierías, Campus León, Universidad de Guanajuato, Loma del Bosque 103, Lomas del Campestre, 37150 León, Gto, Mexico; b Instituto de Física, Benemérita Universidad Autónoma de Puebla, Av. San Claudio y 18 Sur, 72570 Puebla, Pue., Mexico

**Keywords:** crystal structure, coordination compounds, bis­[2-(3,5-di­methyl­pyrazol-1-yl)eth­yl]amine (pza) ligand, bis­(pyrazol-1-yl)amine, platinum(II) complex

## Abstract

The title complex, [PtCl(C_14_H_23_N_5_)]Cl·2H_2_O, is isomorphous with the Pd^II^ compound characterized previously [Mendoza, Bernès & Mendoza-Díaz (2006 ▸). *Acta Cryst*. E**62**, m2934–m2936]. The angle between pyrazole mean planes in the main ligand is 88.3 (4)°, similar to that observed in the Pd^II^ analogue [87.62 (11)°]. This tridentate ligand adopts a conformation approximating a twofold symmetry, allowing its coordination to the metal atom, together with a chloride ligand, in an almost perfect square-planar geometry. A chloride anion and two water mol­ecules in the asymmetric unit form a hydrogen-bonded network connected to the complex mol­ecules in the crystal *via* the NH amine groups, forming chains along [100].

## Related literature   

For the isomorphous Pd^II^ structure, see: Mendoza *et al.* (2006 ▸). For a pseudopolymorph of the Pd^II^ complex, see: Guzei *et al.* (2010 ▸). For other Pd^II^ and Ni^II^ complexes bearing the same bis­(pyrazol-1-yl)amine ligand, see: Mendoza *et al.* (2015 ▸); Ajellal *et al.* (2006 ▸); Massoud *et al.* (2012 ▸, 2013 ▸). 
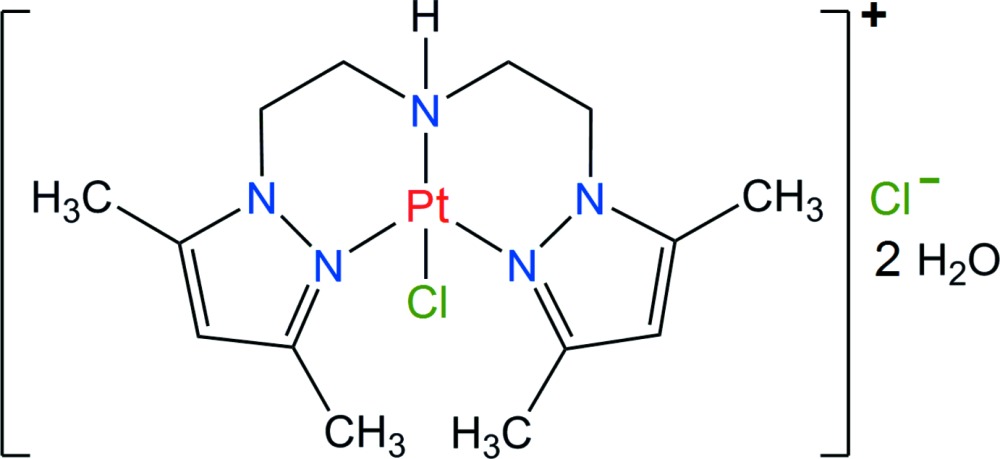


## Experimental   

### Crystal data  

[PtCl(C_14_H_23_N_5_)]Cl·2H_2_O
*M* *_r_* = 563.39
Monoclinic, 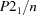

*a* = 7.944 (4) Å
*b* = 22.523 (4) Å
*c* = 11.783 (2) Å
β = 109.34 (2)°
*V* = 1989.1 (11) Å^3^
*Z* = 4
Mo *K*α radiation
μ = 7.34 mm^−1^
*T* = 291 K
0.60 × 0.40 × 0.18 mm

### Data collection  

Bruker P4 diffractometer
Absorption correction: part of the refinement model (Δ*F*) (Walker & Stuart, 1983 ▸) *T*_min_ = 0.024, *T*_max_ = 0.111
4492 measured reflections
3482 independent reflections
3032 reflections with *I* > 2σ(*I*)
*R*_int_ = 0.057
3 standard reflections every 97 reflections intensity decay: 1%

### Refinement  

*R*[*F* ^2^ > 2σ(*F* ^2^)] = 0.052
*wR*(*F* ^2^) = 0.138
*S* = 1.05
3482 reflections
233 parameters
4 restraints
H atoms treated by a mixture of independent and constrained refinement
Δρ_max_ = 2.96 e Å^−3^
Δρ_min_ = −1.26 e Å^−3^

### 

Data collection: *XSCANS* (Siemens, 1996 ▸); cell refinement: *XSCANS*; data reduction: *XSCANS*; program(s) used to solve structure: *SHELXS2014* (Sheldrick, 2008 ▸); program(s) used to refine structure: *SHELXL2014* (Sheldrick, 2015 ▸); molecular graphics: *SHELXTL* (Sheldrick, 2008 ▸) and *Mercury* (Macrae *et al.*, 2008 ▸)’; software used to prepare material for publication: *SHELXL2014*.

## Supplementary Material

Crystal structure: contains datablock(s) I, New_Global_Publ_Block. DOI: 10.1107/S2056989015005307/hp2070sup1.cif

Structure factors: contains datablock(s) I. DOI: 10.1107/S2056989015005307/hp2070Isup2.hkl

**Fig. 1.**
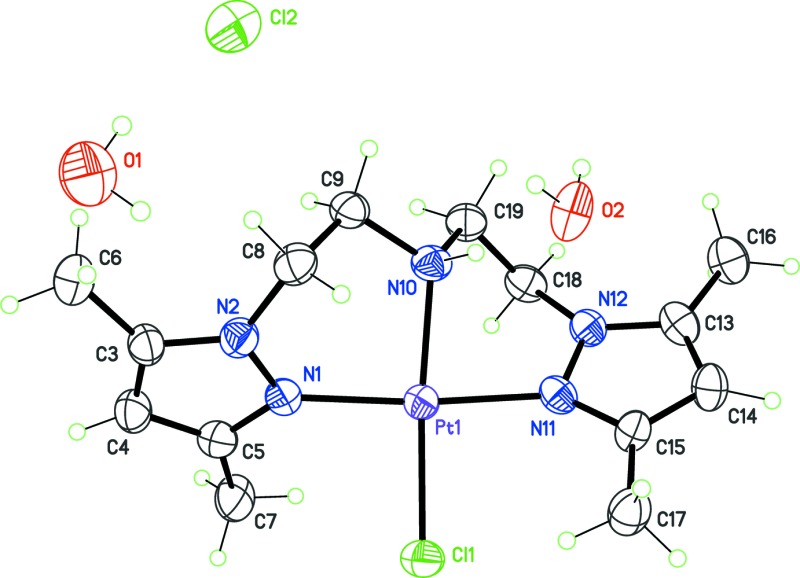
View of the title complex, with displacement ellipsoids for non-H atoms at the 30% probability level.

. DOI: 10.1107/S2056989015005307/hp2070fig1.tif
View of the title complex, with displacement ellipsoids for non-H atoms at the 30% probability level.

**Fig. 2.**
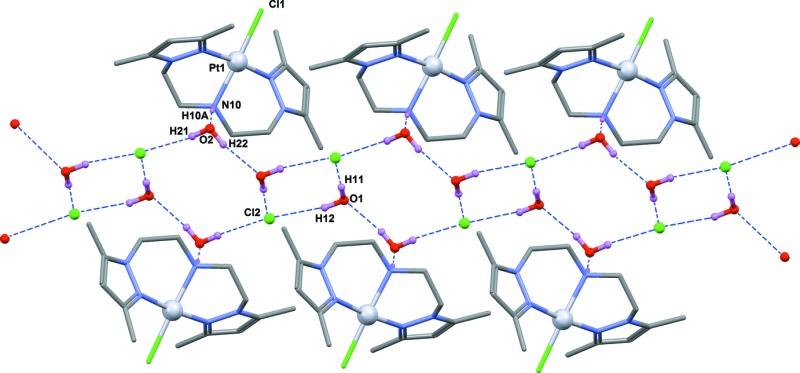
Part of the crystal structure of the title complex, emphasizing the hydrogen-bond network (dashed bonds). H atoms not involved in hydrogen bonds are omitted.

. DOI: 10.1107/S2056989015005307/hp2070fig2.tif
Part of the crystal structure of the title complex, emphasizing the hydrogen-bond network (dashed bonds). H atoms not involved in hydrogen bonds are omitted.

CCDC reference: 1054111

Additional supporting information:  crystallographic information; 3D view; checkCIF report

## Figures and Tables

**Table 1:** Hydrogen-bond geometry (, )

*D*H*A*	*D*H	H*A*	*D* *A*	*D*H*A*
N10H10*A*O2	0.89	2.08	2.960(11)	172
O1H11Cl2^i^	0.85(2)	2.26(3)	3.105(11)	174
O1H12Cl2	0.85(2)	2.28(5)	3.123(11)	169
O2H21Cl2^ii^	0.84(2)	2.24(4)	3.063(10)	166
O2H22O1^i^	0.84(2)	2.01(4)	2.839(13)	169

Symmetry codes: (i) 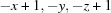; (ii) 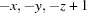.

## supplementary crystallographic information

### S1. Structural commentary

The aim of this structure determination is to confirm that a series of Pd^II^ complexes based on a tridentate ligand, bis­[2-(3,5-di­methyl­pyrazol-1-yl)ethyl]­amine (pza), for which a structural study has been published (Mendoza *et al.*, 2015), is isomorphous to the Pt(II) analogous series. The starting material for this work was a dihydrate, [Pd(pza)Cl]Cl.2 H_2_O, also characterized by X-ray diffraction (Mendoza *et al.*, 2006). However, although a stabilizing hydrogen-bond network is present in this structure, a less hydrated pseudo polymorph, [Pd(pza)Cl]Cl.0.25 H_2_O, has been reported (Guzei *et al.*, 2010). It is unclear whether the difference for water content results from the different starting materials used in the synthesis, Na_2_[PdCl_4_] *vs*. [PdCl_2_(CH_3_CN)_2_], or results from solvents used for crystallization, CH_3_CN *vs*. CH_2_Cl_2_.

The present work reports the characterization of the dihydrate platinum complex, [Pt(pza)Cl]Cl.2 H_2_O (Fig. 1), which is, as expected, isomorphous to its Pd^II^ analogue. Main structural features are thus preserved in the Pt(II) complex: square planar coordination geometry of the metal, *κ*^3^-coordination of the bis­(pyrazol-1-yl)amine ligand, and formation of a stabilizing network of hydrogen bonds, involving two water molecules, the chloride counterion, and the amine NH group of the pza ligand (Fig. 2). Pyrazole mean planes are almost perpendicular to each other, with a dihedral angle of 88.3 (4)°, similar to that observed in the Pd^II^ analogue, 87.62 (11)°.

Inter­estingly, the reported coordination chemistry of this ligand with Ni^II^ is quite different, with regards to structures. In the five-coordinate molecular complexes [Ni(pza)*X*_2_], the pza ligand adopts a flat geometry, characterized by the angle between pyrazole rings of 20.8° (*X* = Cl; Ajellal *et al.*, 2006) or 12.9° (*X* = NCS; Massoud *et al.*, 2012). This arrangement strongly contrasts with that described for a dinuclear six-coordinated Ni^II^ complex, in which pza is folded in order to suit to the o­cta­hedral geometry of the metal. In that case, the dihedral angle between pyrazole rings is 50.7° (Massoud *et al.*, 2013). These structures for 10-group metals show the extreme conformational flexibility of pza, which allows the ligand conformation to be tailored to the requirements of virtually any 4-, 5- or 6-coordinated metal ion.

### S2. Synthesis and crystallization

The synthesis of the Pt(II) complex is parallel to that of the Pd^II^ analogue. K_2_PtCl_4_ (1 mmol) was dissolved in water, and 1 mmol of bis-[2-(3,5-di­methyl-1-pyrazolyl)ethyl]­amine dissolved in hot water was added slowly, under stirring. After 12 h. of stirring at 298 K, a brown solid formed, which was filtered, and dried at 343 K. Yield: 80 %. Elemental analysis of this compound fits for the dihydrated complex crystallized with one KCl molecule: found C 26.57, H 3.86, N 10.55%; calculated for [Pt(C_14_H_23_N_5_)Cl]Cl.2 H_2_O.KCl: C 26.36, H 4.27, N 10.98%. The crude product was redissolved in CH_3_CN, and the precipitate of KCl filtered off. Single crystals of the title compound were obtained after evaporation of CH_3_CN.

### S3. Refinement

In the complex, H atoms were placed in calculated positions and refined with fixed bond lengths, C—H = 0.97, 0.96 and 0.93 Å for methyl­ene, methyl, and aromatic groups respectively, and N—H = 0.89 Å. Water H atoms were found in a difference map and refined with restrained bond lengths, O—H = 0.85 (2) Å. For all H atoms, isotropic displacement parameters were calculated as *U*_iso_(H) = *xU*_eq_(carrier atom), with *x* = 1.5 for methyl CH_3_ and water molecules, and *x* = 1.2 otherwise.

### Crystal data

**Table:**

[PtCl(C_14_H_23_N_5_)]Cl·2H_2_O	*F*(000) = 1096
*M**_r_* = 563.39	*D*_x_ = 1.881 Mg m^−^^3^
Monoclinic, *P*2_1_/*n*	Mo *K*α radiation, λ = 0.71073 Å
*a* = 7.944 (4) Å	Cell parameters from 74 reflections
*b* = 22.523 (4) Å	θ = 4.6–12.5°
*c* = 11.783 (2) Å	µ = 7.34 mm^−^^1^
β = 109.34 (2)°	*T* = 291 K
*V* = 1989.1 (11) Å^3^	Irregular, yellow
*Z* = 4	0.60 × 0.40 × 0.18 mm

### Data collection

**Table:**

Bruker P4 diffractometer	3032 reflections with *I* > 2σ(*I*)
Radiation source: fine-focus sealed tube, FN4	*R*_int_ = 0.057
Graphite monochromator	θ_max_ = 25.0°, θ_min_ = 1.8°
2θ/ω scans	*h* = −9→1
Absorption correction: part of the refinement model (Δ*F*) (Walker & Stuart, 1983)	*k* = −26→1
*T*_min_ = 0.024, *T*_max_ = 0.111	*l* = −13→14
4492 measured reflections	3 standard reflections every 97 reflections
3482 independent reflections	intensity decay: 1%

### Refinement

**Table:**

Refinement on *F*^2^	Primary atom site location: structure-invariant direct methods
Least-squares matrix: full	Secondary atom site location: difference Fourier map
*R*[*F*^2^ > 2σ(*F*^2^)] = 0.052	Hydrogen site location: inferred from neighbouring sites
*wR*(*F*^2^) = 0.138	H atoms treated by a mixture of independent and constrained refinement
*S* = 1.05	*w* = 1/[σ^2^(*F*_o_^2^) + (0.0808*P*)^2^ + 10.4703*P*] where *P* = (*F*_o_^2^ + 2*F*_c_^2^)/3
3482 reflections	(Δ/σ)_max_ = 0.001
233 parameters	Δρ_max_ = 2.96 e Å^−^^3^
4 restraints	Δρ_min_ = −1.26 e Å^−^^3^
0 constraints	

### Fractional atomic coordinates and isotropic or equivalent isotropic displacement parameters (Å^2^)

**Table:**

	*x*	*y*	*z*	*U*_iso_*/*U*_eq_	
Pt1	0.29461 (4)	0.17362 (2)	0.81174 (3)	0.04691 (17)	
Cl1	0.4903 (3)	0.22408 (12)	0.9706 (2)	0.0666 (6)	
Cl2	0.2473 (5)	0.01323 (18)	0.3417 (3)	0.0959 (10)	
N1	0.3971 (10)	0.2093 (3)	0.6922 (7)	0.0529 (17)	
N2	0.4257 (12)	0.1738 (3)	0.6053 (8)	0.0557 (19)	
C3	0.4939 (12)	0.2055 (5)	0.5363 (9)	0.057 (2)	
C4	0.5163 (14)	0.2619 (5)	0.5808 (9)	0.064 (2)	
H4A	0.5659	0.2935	0.5522	0.077*	
C5	0.4526 (13)	0.2634 (4)	0.6751 (9)	0.059 (2)	
C6	0.5244 (18)	0.1798 (5)	0.4275 (11)	0.074 (3)	
H6A	0.5841	0.1423	0.4478	0.111*	
H6B	0.5968	0.2065	0.3999	0.111*	
H6C	0.4118	0.1742	0.3650	0.111*	
C7	0.431 (2)	0.3173 (5)	0.7433 (12)	0.080 (4)	
H7A	0.3608	0.3075	0.7930	0.121*	
H7B	0.3725	0.3480	0.6877	0.121*	
H7C	0.5465	0.3310	0.7930	0.121*	
C8	0.3519 (13)	0.1130 (5)	0.5867 (10)	0.060 (2)	
H8A	0.4091	0.0888	0.6570	0.073*	
H8B	0.3729	0.0949	0.5179	0.073*	
C9	0.1509 (12)	0.1172 (4)	0.5650 (9)	0.055 (2)	
H9A	0.1014	0.1508	0.5134	0.066*	
H9B	0.0923	0.0815	0.5247	0.066*	
N10	0.1180 (10)	0.1241 (3)	0.6811 (7)	0.0542 (18)	
H10A	0.1296	0.0876	0.7115	0.065*	
N11	0.1783 (9)	0.1329 (3)	0.9188 (7)	0.0512 (17)	
N12	−0.0039 (10)	0.1288 (3)	0.8745 (7)	0.0530 (17)	
C13	−0.0607 (13)	0.0933 (4)	0.9487 (10)	0.060 (2)	
C14	0.0871 (13)	0.0748 (5)	1.0375 (10)	0.064 (3)	
H14A	0.0893	0.0502	1.1013	0.077*	
C15	0.2331 (12)	0.0988 (4)	1.0167 (8)	0.053 (2)	
C16	−0.2500 (13)	0.0794 (5)	0.9250 (11)	0.071 (3)	
H16A	−0.3143	0.1154	0.9261	0.107*	
H16B	−0.2613	0.0529	0.9860	0.107*	
H16C	−0.2982	0.0609	0.8476	0.107*	
C17	0.4253 (14)	0.0902 (6)	1.0886 (10)	0.071 (3)	
H17A	0.4955	0.1200	1.0669	0.106*	
H17B	0.4632	0.0516	1.0724	0.106*	
H17C	0.4409	0.0935	1.1727	0.106*	
C18	−0.1019 (13)	0.1636 (4)	0.7703 (10)	0.060 (2)	
H18A	−0.0629	0.2047	0.7823	0.072*	
H18B	−0.2283	0.1624	0.7596	0.072*	
C19	−0.0704 (12)	0.1391 (5)	0.6588 (9)	0.064 (3)	
H19A	−0.1426	0.1038	0.6322	0.077*	
H19B	−0.1081	0.1683	0.5948	0.077*	
O1	0.6546 (14)	0.0365 (5)	0.3986 (10)	0.093 (3)	
H11	0.68 (2)	0.026 (8)	0.471 (6)	0.139*	
H12	0.543 (5)	0.030 (8)	0.373 (17)	0.139*	
O2	0.1596 (12)	−0.0015 (4)	0.7589 (8)	0.076 (2)	
H21	0.049 (5)	−0.001 (7)	0.722 (13)	0.114*	
H22	0.205 (17)	−0.016 (7)	0.710 (10)	0.114*	

### Atomic displacement parameters (Å^2^)

**Table:**

	*U*^11^	*U*^22^	*U*^33^	*U*^12^	*U*^13^	*U*^23^
Pt1	0.0457 (2)	0.0476 (2)	0.0468 (3)	−0.00155 (12)	0.01444 (16)	0.00047 (13)
Cl1	0.0658 (14)	0.0665 (15)	0.0609 (14)	−0.0101 (12)	0.0120 (11)	−0.0062 (12)
Cl2	0.099 (2)	0.111 (3)	0.0747 (19)	−0.0099 (19)	0.0257 (17)	−0.0069 (18)
N1	0.054 (4)	0.055 (4)	0.052 (4)	−0.006 (3)	0.020 (3)	−0.003 (3)
N2	0.062 (5)	0.055 (5)	0.052 (5)	−0.001 (3)	0.022 (4)	−0.002 (3)
C3	0.050 (5)	0.067 (6)	0.056 (5)	−0.004 (4)	0.021 (4)	0.006 (5)
C4	0.067 (6)	0.067 (6)	0.061 (6)	−0.004 (5)	0.023 (5)	0.013 (5)
C5	0.059 (5)	0.059 (6)	0.058 (6)	−0.006 (4)	0.016 (4)	−0.003 (4)
C6	0.079 (7)	0.083 (8)	0.069 (7)	0.001 (6)	0.037 (6)	0.004 (6)
C7	0.126 (11)	0.046 (6)	0.078 (8)	−0.017 (6)	0.044 (8)	0.000 (5)
C8	0.068 (6)	0.060 (6)	0.058 (6)	0.002 (5)	0.027 (5)	−0.009 (5)
C9	0.061 (5)	0.048 (5)	0.053 (5)	−0.007 (4)	0.014 (4)	−0.002 (4)
N10	0.063 (5)	0.042 (4)	0.054 (5)	−0.002 (3)	0.014 (3)	−0.005 (3)
N11	0.045 (4)	0.048 (4)	0.063 (5)	−0.001 (3)	0.022 (3)	−0.002 (3)
N12	0.047 (4)	0.056 (4)	0.057 (5)	−0.002 (3)	0.019 (3)	0.000 (4)
C13	0.055 (5)	0.052 (5)	0.083 (7)	0.001 (4)	0.035 (5)	−0.004 (5)
C14	0.064 (6)	0.074 (7)	0.067 (6)	0.002 (5)	0.039 (5)	0.008 (5)
C15	0.055 (5)	0.061 (6)	0.047 (5)	0.006 (4)	0.021 (4)	0.007 (4)
C16	0.057 (6)	0.073 (7)	0.092 (8)	0.006 (5)	0.037 (6)	0.005 (6)
C17	0.059 (6)	0.089 (8)	0.063 (6)	0.008 (5)	0.019 (5)	0.016 (6)
C18	0.047 (5)	0.056 (5)	0.071 (7)	0.003 (4)	0.011 (5)	0.000 (5)
C19	0.045 (5)	0.081 (7)	0.058 (6)	−0.004 (5)	0.005 (4)	0.007 (5)
O1	0.095 (6)	0.083 (6)	0.104 (7)	0.008 (5)	0.037 (6)	0.015 (5)
O2	0.085 (5)	0.084 (5)	0.069 (5)	0.028 (4)	0.042 (4)	0.025 (4)

### Geometric parameters (Å, º)

**Table:**

Pt1—N1	2.013 (7)	N10—H10A	0.8900
Pt1—N11	2.015 (7)	N11—C15	1.332 (12)
Pt1—N10	2.037 (7)	N11—N12	1.370 (10)
Pt1—Cl1	2.298 (3)	N12—C13	1.366 (12)
N1—C5	1.334 (12)	N12—C18	1.448 (13)
N1—N2	1.375 (11)	C13—C14	1.354 (15)
N2—C3	1.325 (12)	C13—C16	1.470 (14)
N2—C8	1.477 (13)	C14—C15	1.373 (13)
C3—C4	1.364 (15)	C14—H14A	0.9300
C3—C6	1.497 (15)	C15—C17	1.493 (14)
C4—C5	1.365 (14)	C16—H16A	0.9600
C4—H4A	0.9300	C16—H16B	0.9600
C5—C7	1.497 (15)	C16—H16C	0.9600
C6—H6A	0.9600	C17—H17A	0.9600
C6—H6B	0.9600	C17—H17B	0.9600
C6—H6C	0.9600	C17—H17C	0.9600
C7—H7A	0.9600	C18—C19	1.520 (16)
C7—H7B	0.9600	C18—H18A	0.9700
C7—H7C	0.9600	C18—H18B	0.9700
C8—C9	1.534 (13)	C19—H19A	0.9700
C8—H8A	0.9700	C19—H19B	0.9700
C8—H8B	0.9700	O1—H11	0.85 (2)
C9—N10	1.483 (12)	O1—H12	0.85 (2)
C9—H9A	0.9700	O2—H21	0.84 (2)
C9—H9B	0.9700	O2—H22	0.84 (2)
N10—C19	1.470 (12)		
			
N1—Pt1—N11	174.6 (3)	C19—N10—Pt1	114.5 (6)
N1—Pt1—N10	91.5 (3)	C9—N10—Pt1	117.9 (6)
N11—Pt1—N10	83.1 (3)	C19—N10—H10A	104.3
N1—Pt1—Cl1	92.9 (2)	C9—N10—H10A	104.3
N11—Pt1—Cl1	92.4 (2)	Pt1—N10—H10A	104.3
N10—Pt1—Cl1	175.3 (2)	C15—N11—N12	107.0 (7)
C5—N1—N2	105.9 (7)	C15—N11—Pt1	135.9 (6)
C5—N1—Pt1	134.5 (7)	N12—N11—Pt1	116.0 (6)
N2—N1—Pt1	119.6 (6)	C13—N12—N11	109.1 (8)
C3—N2—N1	110.2 (7)	C13—N12—C18	131.4 (8)
C3—N2—C8	129.1 (9)	N11—N12—C18	119.3 (7)
N1—N2—C8	119.6 (8)	C14—C13—N12	106.8 (8)
N2—C3—C4	107.0 (9)	C14—C13—C16	130.7 (10)
N2—C3—C6	122.2 (9)	N12—C13—C16	122.5 (10)
C4—C3—C6	130.6 (9)	C13—C14—C15	108.0 (9)
C3—C4—C5	107.5 (9)	C13—C14—H14A	126.0
C3—C4—H4A	126.3	C15—C14—H14A	126.0
C5—C4—H4A	126.3	N11—C15—C14	109.1 (9)
N1—C5—C4	109.3 (9)	N11—C15—C17	123.0 (8)
N1—C5—C7	123.9 (9)	C14—C15—C17	127.8 (9)
C4—C5—C7	126.6 (10)	C13—C16—H16A	109.5
C3—C6—H6A	109.5	C13—C16—H16B	109.5
C3—C6—H6B	109.5	H16A—C16—H16B	109.5
H6A—C6—H6B	109.5	C13—C16—H16C	109.5
C3—C6—H6C	109.5	H16A—C16—H16C	109.5
H6A—C6—H6C	109.5	H16B—C16—H16C	109.5
H6B—C6—H6C	109.5	C15—C17—H17A	109.5
C5—C7—H7A	109.5	C15—C17—H17B	109.5
C5—C7—H7B	109.5	H17A—C17—H17B	109.5
H7A—C7—H7B	109.5	C15—C17—H17C	109.5
C5—C7—H7C	109.5	H17A—C17—H17C	109.5
H7A—C7—H7C	109.5	H17B—C17—H17C	109.5
H7B—C7—H7C	109.5	N12—C18—C19	109.9 (8)
N2—C8—C9	108.0 (8)	N12—C18—H18A	109.7
N2—C8—H8A	110.1	C19—C18—H18A	109.7
C9—C8—H8A	110.1	N12—C18—H18B	109.7
N2—C8—H8B	110.1	C19—C18—H18B	109.7
C9—C8—H8B	110.1	H18A—C18—H18B	108.2
H8A—C8—H8B	108.4	N10—C19—C18	112.2 (8)
N10—C9—C8	110.1 (8)	N10—C19—H19A	109.2
N10—C9—H9A	109.6	C18—C19—H19A	109.2
C8—C9—H9A	109.6	N10—C19—H19B	109.2
N10—C9—H9B	109.6	C18—C19—H19B	109.2
C8—C9—H9B	109.6	H19A—C19—H19B	107.9
H9A—C9—H9B	108.2	H11—O1—H12	101 (10)
C19—N10—C9	109.8 (7)	H21—O2—H22	106 (10)
			
C5—N1—N2—C3	−1.0 (11)	C15—N11—N12—C13	2.0 (10)
Pt1—N1—N2—C3	−179.8 (6)	Pt1—N11—N12—C13	171.7 (6)
C5—N1—N2—C8	−170.2 (9)	C15—N11—N12—C18	177.2 (8)
Pt1—N1—N2—C8	10.9 (11)	Pt1—N11—N12—C18	−13.1 (10)
N1—N2—C3—C4	2.2 (11)	N11—N12—C13—C14	−1.1 (11)
C8—N2—C3—C4	170.1 (10)	C18—N12—C13—C14	−175.5 (10)
N1—N2—C3—C6	−174.6 (9)	N11—N12—C13—C16	−179.3 (9)
C8—N2—C3—C6	−6.7 (17)	C18—N12—C13—C16	6.3 (16)
N2—C3—C4—C5	−2.5 (12)	N12—C13—C14—C15	−0.3 (12)
C6—C3—C4—C5	173.9 (11)	C16—C13—C14—C15	177.7 (11)
N2—N1—C5—C4	−0.6 (11)	N12—N11—C15—C14	−2.2 (11)
Pt1—N1—C5—C4	177.9 (7)	Pt1—N11—C15—C14	−168.8 (7)
N2—N1—C5—C7	174.4 (11)	N12—N11—C15—C17	178.0 (9)
Pt1—N1—C5—C7	−7.0 (16)	Pt1—N11—C15—C17	11.4 (16)
C3—C4—C5—N1	1.9 (12)	C13—C14—C15—N11	1.6 (12)
C3—C4—C5—C7	−172.9 (11)	C13—C14—C15—C17	−178.7 (11)
C3—N2—C8—C9	−112.7 (11)	C13—N12—C18—C19	−115.1 (11)
N1—N2—C8—C9	54.3 (11)	N11—N12—C18—C19	71.0 (11)
N2—C8—C9—N10	−79.8 (10)	C9—N10—C19—C18	−163.9 (8)
C8—C9—N10—C19	169.6 (8)	Pt1—N10—C19—C18	−28.6 (11)
C8—C9—N10—Pt1	36.0 (10)	N12—C18—C19—N10	−43.4 (12)

### Hydrogen-bond geometry (Å, º)

**Table:**

*D*—H···*A*	*D*—H	H···*A*	*D*···*A*	*D*—H···*A*
N10—H10*A*···O2	0.89	2.08	2.960 (11)	172
O1—H11···Cl2^i^	0.85 (2)	2.26 (3)	3.105 (11)	174
O1—H12···Cl2	0.85 (2)	2.28 (5)	3.123 (11)	169
O2—H21···Cl2^ii^	0.84 (2)	2.24 (4)	3.063 (10)	166
O2—H22···O1^i^	0.84 (2)	2.01 (4)	2.839 (13)	169

Symmetry codes: (i) −*x*+1, −*y*, −*z*+1; (ii) −*x*, −*y*, −*z*+1.
