# Supplementary figures and images for: Crystal structure of {bis­[2-(3,5-di­methyl­pyrazol-1-yl-κN 2)eth­yl]amine-κN}chlorido­platinum(II) chloride dihydrate
Source: Acta Crystallogr E Crystallogr Commun. 2015 Mar 21;71(Pt 4):m98–9. doi: 10.1107/S2056989015005307 (PMC4438831; doi:10.1107/S2056989015005307)

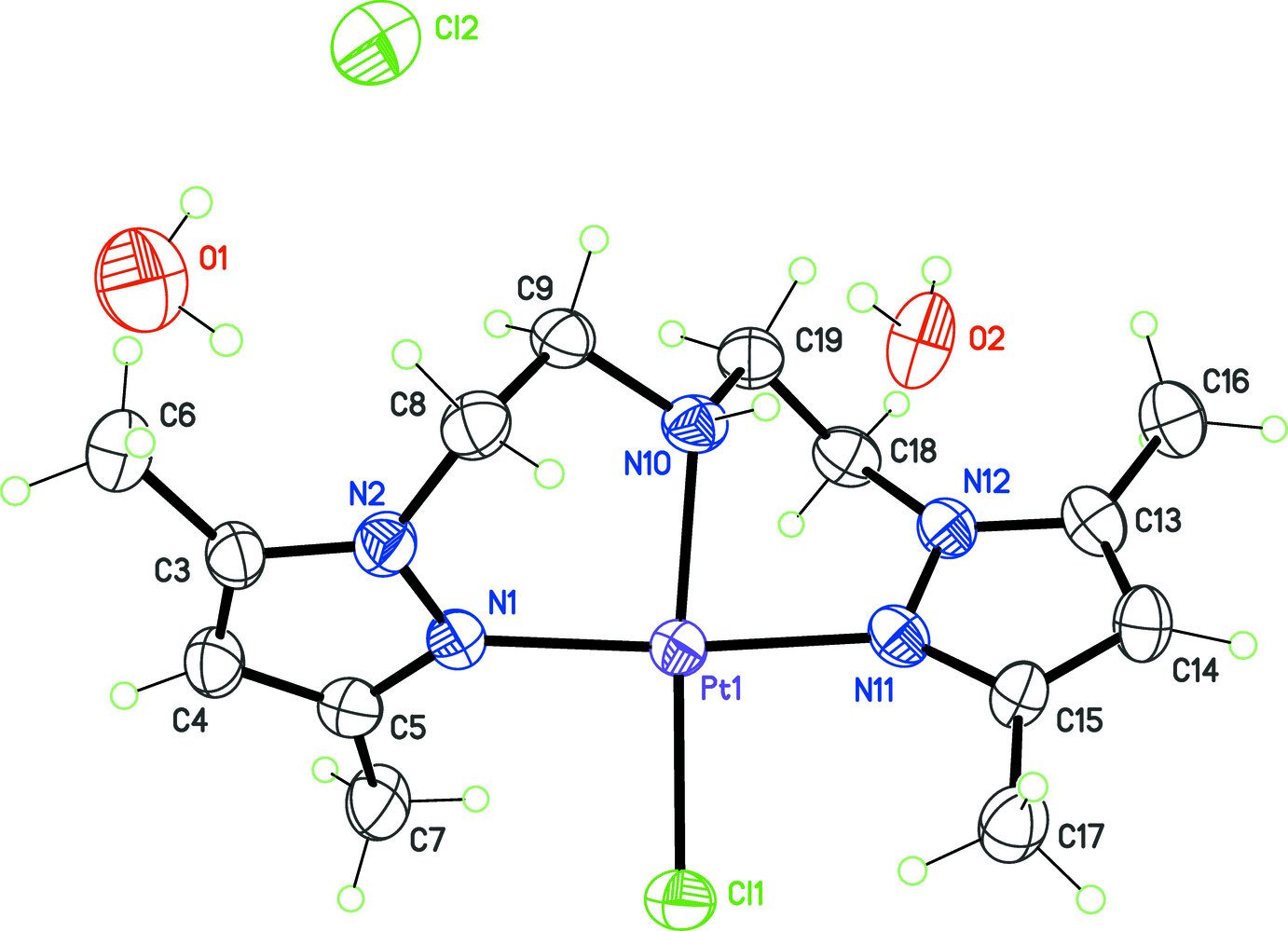

Supplement: Supplementary file 3 [file e-71-00m98-fig1.tif]

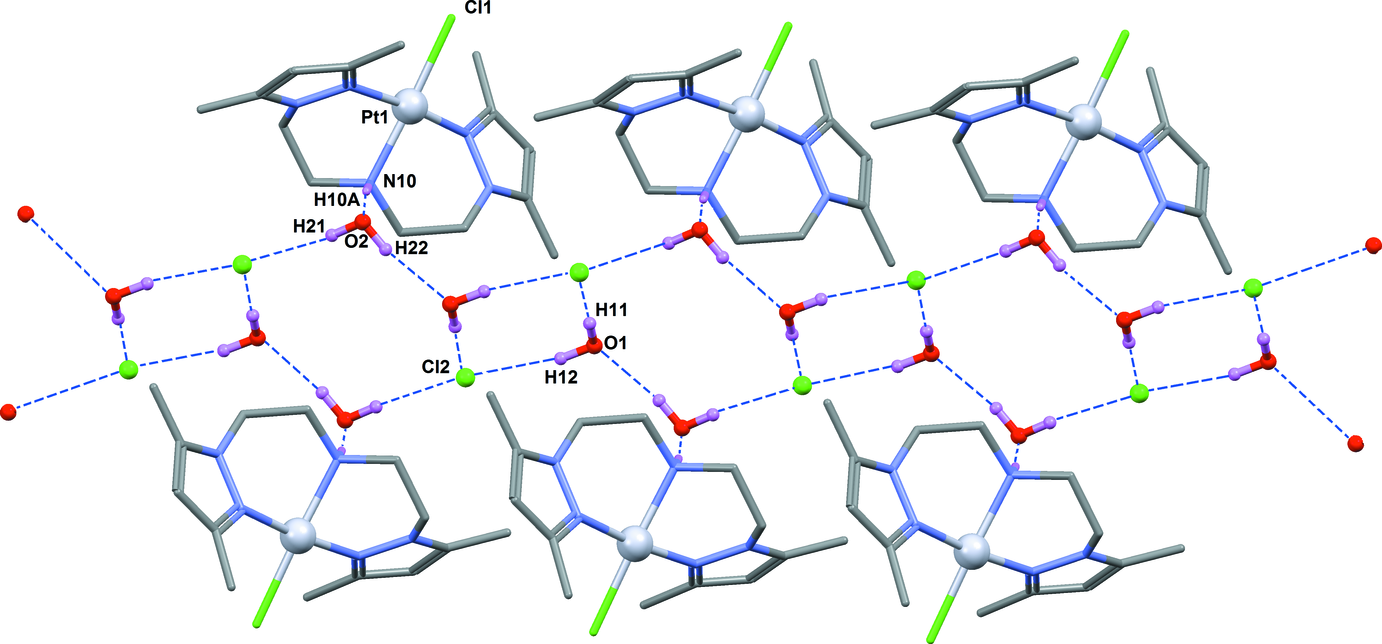

Supplement: Supplementary file 4 [file e-71-00m98-fig2.tif]
